# Association between maternal polycystic ovary syndrome and attention-deficit/hyperactivity disorder in offspring aged 3–6 years: A Chinese population-based study

**DOI:** 10.3389/fpubh.2022.1032315

**Published:** 2023-01-09

**Authors:** Yuying Zhang, Dali Lu, Vivian Yawei Guo, Yuqing Wang, Shuangyan Qiu, Jingyu Zhang, Yan Zhang, Weiqing Chen, Baoping Wang, Weikang Yang

**Affiliations:** ^1^Department of Child Healthcare, Shenzhen Longhua Maternity and Child Healthcare Hospital, Shenzhen, China; ^2^Department of Epidemiology, School of Public Health, Sun Yat-sen University, Guangzhou, China; ^3^Health Management Center, Guangzhou First People's Hospital, Guangzhou, China; ^4^Department of Infertility and Reproductive Medicine, Shenzhen Longhua Maternity and Child Healthcare Hospital, Shenzhen, China

**Keywords:** maternal polycystic ovary syndrome (PCOS), attention-deficit/hyperactivity disorder (ADHD), offspring, sex difference, treatment

## Abstract

**Background:**

Maternal polycystic ovary syndrome (PCOS) may increase the risk of attention-deficit/hyperactivity disorder (ADHD) in offspring; however, their association remains unexplored in Asian populations. Hence, this study aimed to investigate the association between maternal PCOS and ADHD in offspring aged 3–6 years and whether it differed by offspring sex.

**Methods:**

This was a district-wide population-based study of 87,081 preschoolers from 234 kindergartens in Longhua District, Shenzhen, China. The parents were invited to complete a self-administrated questionnaire covering information on socio-demographics, maternal disease history, and child behavior. ADHD symptoms were measured with the parent-rating 26-item Swanson, Nolan, and Pelham Rating Scale (SNAP-IV). Logistic regression was performed to examine the associations between maternal PCOS and ADHD symptoms in offspring.

**Results:**

The response rate was 80% and 63,390 mother-child pairs were included. Of the mothers, 1,667 (2.6%) reported PCOS diagnoses. The mean age of children at ADHD assessment was 4.86 ± 0.84[SD] years, and 53.6% were boys. Children with maternal PCOS had a higher risk of developing ADHD symptoms than other children (12.0 vs. 9.4%, adjusted odds ratio [OR] = 1.32, 95% CI: 1.13–1.54). The risk estimate was significant in boys (adjusted OR = 1.38, 95% CI: 1.14–1.66) but not in girls (adjusted OR = 1.23, 95% CI: 0.94–1.57, *P* for interaction = 0.391). Treatment of PCOS tended to be associated with a lower risk of ADHD symptoms than untreated PCOS albeit risk confidence intervals were overlapped (treated: adjusted OR = 1.28, 95% CI: 1.06–1.54 vs. untreated: adjusted OR = 1.14, 95% CI: 1.08–1.83).

**Conclusion:**

Maternal PCOS increases the risk of developing ADHD in offspring, especially boys. Further studies are warranted to confirm our findings, and early neurodevelopmental screening may be needed in children born to mothers with PCOS.

## Introduction

Attention-deficit/hyperactivity disorder (ADHD) is the most common neurodevelopmental disorder in children, with an estimated prevalence of 2–7% globally (1, 2). Approximately 40–60% of childhood ADHD will persist into adulthood and increase the risk of other mental health disorders, posing lifelong challenges to individuals and families (3–5). The exact mechanisms underlying ADHD remain elusive, but there is growing evidence supporting the crucial gene-environment interactions in the development of ADHD (5–7).

Polycystic ovary syndrome (PCOS) is the most common endocrine disorder, affecting 6–20% of women of reproductive age (8). PCOS is characterized by hyperandrogenism, with clinical manifestations of irregular menstruation, hirsutism, and infertility. High levels of circulating androgen exposure may affect fetal brain development during pregnancy (9). As a result, there is growing awareness that PCOS may increase the risk of neurodevelopmental disorders in offspring (10–16). For example, a national registry-based study in Sweden reported 42% greater odds of offspring ADHD associated with maternal PCOS (10). A similar risk estimate (42%) for ADHD has been reported in a Finnish population-based cohort study. Children born to mothers with PCOS have an increased risk of psychiatric diagnoses, including ADHD (14). However, these studies mainly involved Caucasians, and the association between maternal PCOS and offspring ADHD in the Asian population remains unexplored. Although ADHD appears to be a universal syndrome, the perceptions, diagnosis, and treatment of the disorder are deeply influenced by cultures (17). Therefore, investigating these associations in the Chinese cultural context is valuable.

Although ADHD predominantly occurs in males (1), it remains unclear whether boys are more susceptible to maternal PCOS exposure than girls (13–15). For example, two Swedish studies reported a stronger association between maternal PCOS and offspring ADHD in girls than in boys (10, 13). In contrast, the Odense Child Cohort in Denmark reported a significant correlation between maternal PCOS and ADHD symptoms in boys but not in girls (15). Therefore, further studies are required to clarify sex differences in the association between maternal PCOS and offspring ADHD. Moreover, few studies have examined the associations of maternal PCOS treatment with ADHD in offspring. However, emerging evidence suggests that some commonly prescribed medicines for PCOS, such as hormonal contraceptives, were associated with an increased risk of ADHD in the offspring (18).

To this end, this study aimed to investigate the association between maternal self-reported PCOS and offspring ADHD among 3- to 6-year-old Chinese preschoolers in a population-based study. We also performed a stratified analysis to test if the association differed according to offspring sex and PCOS treatment status.

## Materials and methods

### Study population

The Longhua Child Cohort Study (LCCS) is an ongoing district-wide population-based study to evaluate the impact of early life family and school environments on preschoolers' psycho-behavioral development. First initiated in 2014, the LCCS has been performed annually among kindergarten children in the Longhua District of Shenzhen in mainland China. This study was based on a 2021 wave survey involving 234 kindergartens conducted between November and December 2021. Parents were invited to complete a structured questionnaire assigned through a mobile app. The questionnaire includes information on socio-demographics, maternal condition during pregnancy (e.g., pregnancy complications, weight gain, smoking exposure), children's characteristics, and early-life exposure at age 0–1 and age 1–3 years (e.g., birthweight, feeding pattern, outdoor activity), as well as children's psycho-behavioral conditions assessed by validated questionnaires. The details of the study design and implementation procedures of the LCCS have been described in previous studies (19–21).

**Figure 1:**
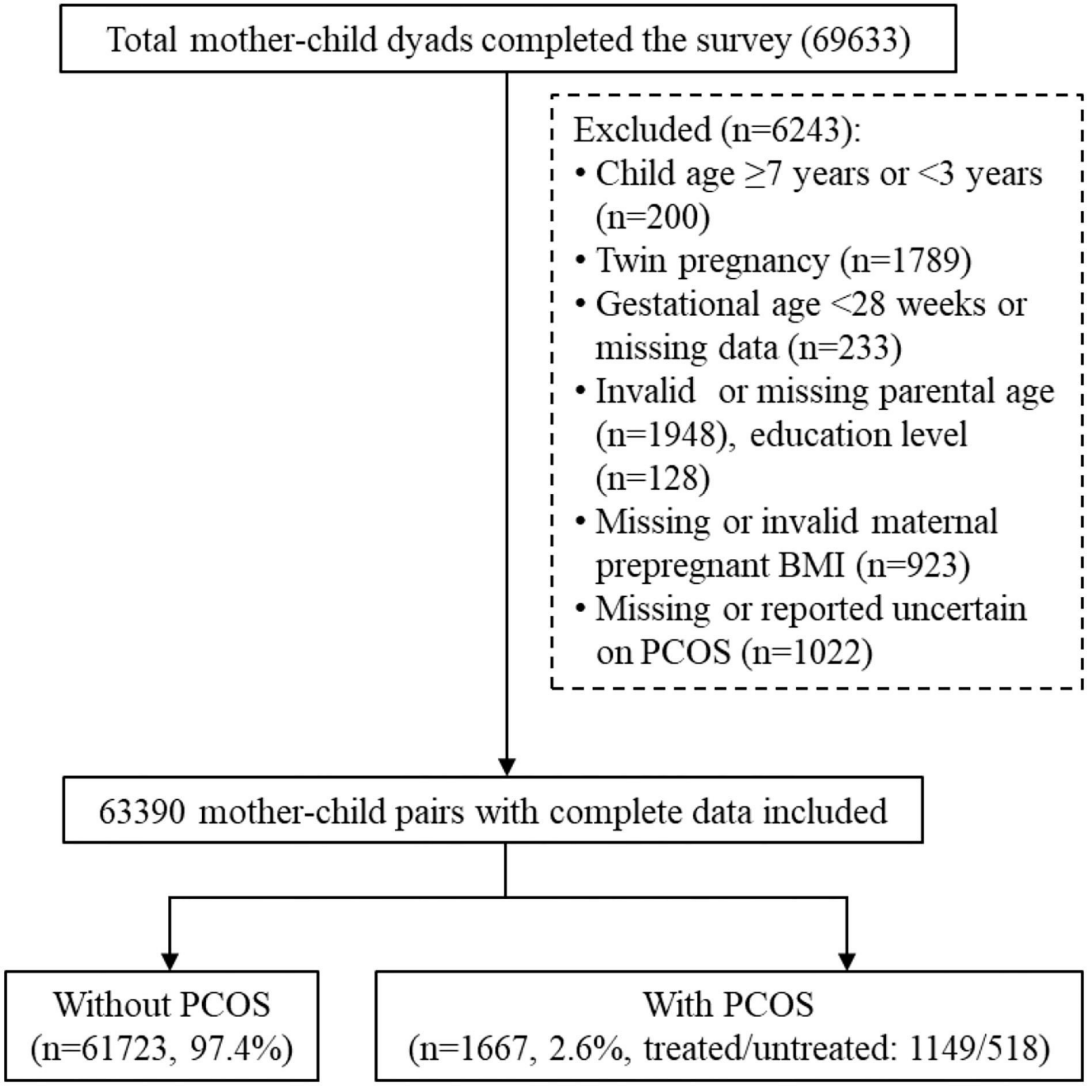
Flowchart of participant selection. PCOS, polycystic ovary syndrome; BMI, body mass index; GDM, gestational diabetes mellitus.

During the study period, 87,081 preschoolers were attending 234 kindergartens in Longhua District. Among them, 69,633 mother-child pairs completed the questionnaires, with a response rate of 80%. After excluding children under 3 years and above 6 years or those with missing or invalid data on maternal PCOS and covariates, 63,390 mother-child pairs were included in the current analysis. The data selection procedure is illustrated in Figure 1. This study was approved by the Ethics Committee of Shenzhen Longhua Maternity and Child Healthcare Hospital, and informed consent was obtained from all participants.

### Measurement of PCOS exposure

History of PCOS was assessed using a self-reported question, “have you ever been diagnosed with PCOS before this pregnancy?” The answers to this question were “no,” “yes, but not treated,” “yes, treated,” and “uncertain.” A mother was considered as having a history of PCOS if she had reported “yes” regardless of treatment status. In the current study, participants who reported “uncertain” were excluded from the analysis.

### Measurement of ADHD symptoms at age 3–6 years

The widely used 26-item Parent-rating Swanson, Nolan, and Pelham (SNAP) revision 4 (SNAP-IV) scale was adopted to assess ADHD symptoms (22, 23). The Chinese SNAP-IV demonstrated good internal reliability (Cronbach's α = 0.88–0.90) and satisfactory test-retest reliability (intraclass correlation = 0.59–0.72) in a previous study (22). The SNAP-IV scale had excellent reliability in this study, with a Cronbach's alpha coefficient of 0.92. This scale is based on a 0 to 3 rating scale from “not at all” to “very often.” Two subsets of symptoms derived from the Diagnostic and Statistical Manual of Mental Disorders (DSM-IV) criteria for ADHD were included: inattention (items 1–9) and hyperactivity/impulsivity (Items 10–18). Defiant disorder (ODD) is often present in children with ADHD; hence, the SNAP-IV scale also included the opposition/defiance subset (items 19–26) derived from the DSM-IV criteria for ODD diagnosis. The subset scores were calculated by summing the scores of the items of each subset. A child was defined as having borderline symptoms in the subset if they had at least mild symptoms of inattention (subset score ≥ 13), hyperactivity/impulsivity (subset score ≥ 13), and opposition/defiance (subset score ≥ 8). ADHD was defined as an inattention score of ≥13 or a hyperactivity/impulsivity score of ≥13.

### Statistical analysis

Two-sample independent *t*-tests and χ^2^ tests were used to compare the maternal, paternal, and child characteristics of those with or without maternal PCOS exposure. A logistic regression model was used to evaluate the association between maternal PCOS history and offspring ADHD borderline symptoms. Covariates related to socio-demographics and environmental exposures suggested to play important roles in ADHD development were included in the logistic regression models. These include maternal age at childbirth, education, marital status, household income, passive smoking during pregnancy, and pre-pregnant body mass index [BMI]); paternal age at childbirth, education; and child sex and age at ADHD assessment (24–27). These covariates were captured using a self-reported questionnaire. In this study, we collected data on passive but not active smoking because active smoking during pregnancy was rare compared to passive smoking. Besides, we did not adjust other child characteristics (e.g., conception with infertility treatment, mode of delivery, gestational weeks, and birth weight) because they occur after the exposure and are likely consequences of PCOS and may be mediators on the causal pathway. Adjustment for mediators does not reveal the direct effect of an exposure (e.g., PCOS) on an outcome (e.g., child behavior) and can introduce bias (11, 28). We also performed sensitivity analysis by treating SNAP-IV total score as the ADHD severity outcome using general linear regression model.

The *P*-value for interaction was calculated by introducing an interaction variable “PCOS^*^child sex” in the regression model to evaluate child sex differences in the impact of maternal PCOS on offspring ADHD. We also performed subgroup analysis by dividing the participants into two subgroups according to the child's sex. All statistical analyses were performed using R software (version 4.2), and a *P*-value of <0.05 (two-sided) was considered statistically significant.

## Results

### Participant characteristics

The mean maternal age of childbirth was 29.1 ± 4.4 years, and more than 65.2% of parents received at least college education. The mean child age at ADHD assessment was 4.86 ± 0.84 years, and 53.6% of children were boys. Overall, 1,667 (2.6%) mothers reported a history of PCOS, of which 1,149 (68.9%) were treated for PCOS. Mothers with self-reported PCOS were older at childbirth, more educated, and had higher household income and higher pre-pregnant BMI than their counterparts. Additionally, children born to mothers with PCOS had a shorter gestational age and lower birthweight and were more likely to be born with fertility treatment and cesarean delivery. The characteristics of the study participants are presented in Table 1.

**Table 1:** Comparison of demographic and clinical characteristics between the PCOS and non-PCOS groups.

	**Total (*n* = 63,390)**	**Non-PCO (*n* = 61,723)**	**PCOS (*n* = 1,667)**	***P*-value**
**Parental characteristics**				
Maternal age at childbirth (years)	29.1 ± 4.4	29.1 ± 4.4	29.4 ± 3.7	0.001
**Maternal education**, ***n*** **(%)**				
Middle school or less	9,266 (14.6)	9,106 (14.8)	160 (9.6)	< 0.001
High school	12,822 (20.2)	12,556 (20.3)	266 (16.0)	
College or above	41,302 (65.2)	40,061 (64.9)	1,241 (74.4)	
**Marital status**, ***n*** **(%)**				
Married	62,783 (99.0)	61,133 (99.0)	1,650 (99.0)	0.891
Unmarried/Divorced/Windowed	607 (1.0)	590 (1.0)	17 (1.0)	
**Household income, RMB/month**, ***n*** **(%)**				
< 10,000	9,390 (14.8)	9,219 (14.9)	171 (10.3)	< 0.001
10,000–20,000	21,933 (34.6)	21,418 (34.7)	515 (30.9)	
20,000–30,000	13,851 (21.9)	13,458 (21.8)	393 (23.6)	
≥300,000	18,216 (28.7)	17,628 (28.6)	588 (35.3)	
Maternal exposure to second-hand smoke, *n* (%)	11,003 (17.4)	10,713 (17.4)	290 (17.4)	0.992
Paternal age at childbirth (years)	31.2 ± 5.10	31.2 ± 5.1	31.5 ± 4.8	0.007
**Paternal education**, ***n*** **(%)**				
Middle school or less	8,289 (13.1)	8,133 (13.2)	156 (9.4)	< 0.001
High school	13,016 (20.5)	12,742 (20.6)	274 (16.4)	
College or above	42,085 (66.4)	40,848 (66.2)	1,237 (74.2)	
Maternal prepregnant BMI (kg/m^2^)	20.9 ± 2.9	20.8 ± 2.9	21.7 ± 3.2	< 0.001
**Child Characteristics**				
Child age at assessment (years)	4.86 ± 0.84	4.86 ± 0.84	4.71 ± 0.86	< 0.001
**Sex**, ***n*** **(%)**				
Boy	33,956 (53.6)	33,065 (53.6)	891 (53.4)	0.942
Girl	29,434 (46.4)	28,658 (46.4)	776 (46.6)	
Conception with fertility treatment	970 (1.5)	699 (1.1)	271 (16.3)	< 0.001
**Delivery mode**, ***n*** **(%)**				
Eutocia	42,437 (66.9)	41,387 (67.1)	1,050 (63.0)	0.001
Cesarean	20,953 (33.1)	20,336 (32.9)	617 (37.0)	
Gestational weeks	39.0 ± 1.6	39.0 ± 1.6	38.9 ± 1.8	0.073
Birth weight (kg)	3.22 ± 0.46	3.22 ± 0.46	3.20 ± 0.49	0.029
SNAP-IV total score	17 (11–24)	17 (11–24)	19 (12.25)	< 0.001
ADHD symptoms	6,005 (9.5)	5,805 (9.4)	200 (12.0)	< 0.001
**Borderline symptoms** ^*^				
Inattention	3,469 (5.5)	3,339 (5.4)	130 (7.8)	< 0.001
Hyperactivity/impulsivity	4,289 (6.8)	4,145 (6.7)	144 (8.6)	0.002
Opposition/defiance	14,873 (23.5)	14,351 (23.3)	522 (31.3)	< 0.001

Data were expressed as mean ± standard deviation, number (%), or median (interquartile range).

PCOS, polycystic ovary syndrome; BMI, body mass index; ADHD, attention-deficit/hyperactivity disorder.

* A child was defined as having borderline symptoms on the subset if he/she had at least mild symptoms of inattention (subset score ≥ 13), hyperactivity/impulsivity (subset score ≥ 13), and opposition/defiance (subset score ≥ 8). ADHD was defined if the inattention score ≥ 13 and/or hyperactivity/impulsivity score ≥ 13.

### Risk for ADHD symptoms in PCOS-exposed offspring aged 3–6 years

Overall, 9.5% of the children had ADHD symptoms, which was higher in children with maternal PCOS than in their counterparts (12.0 vs. 9.4%, *P* < 0.001). Specifically, 7.8, 8.6, and 31.3% of children with maternal PCOS exposure were identified as having borderline symptoms of inattention, hyperactivity/impulsivity, and opposition/defiance, respectively, compared to 5.4, 6.7, and 23.3% of those without PCOS exposure (Table 1).

In the crude model, children born to mothers with PCOS were at a higher risk of having symptoms of ADHD (OR = 1.30, 95% CI: 1.13–1.53), inattention (OR = 1.48, 95% CI: 1.23–1.77), hyperactivity/impulsivity (OR = 1.31, 95% CI: 1.10–1.56), and opposition/defiance (OR = 1.51, 95% CI: 1.35–1.67) compared to those without PCOS exposure. Even after controlling for confounding variables, maternal PCOS was still associated with an increased risk of ADHD (adjusted OR = 1.32, 95% CI: 1.13–1.54) and borderline symptoms of inattention (adjusted OR = 1.53, 95% CI: 1.27–1.83), hyperactivity/impulsivity (adjusted OR = 1.31, 95% CI: 1.09–1.55), and opposition/defiance (adjusted OR = 1.46, 95% CI: 1.31–1.62) (Table 2). Similarly, children born to mothers with PCOS were associated with an increased SNAP score in both the crude model (β = 1.94, 95% CI: 1.46–2.42) and adjusted model (β = 1.91, 95% CI: 1.44–2.39) (Supplementary Table S1).

**Table 2:** Odds of maternal PCOS for ADHD and borderline symptoms in offspring aged 3–6 years^*^.

	**Crude model**		**Adjusted model** ^**¶**^	
	**OR (95% CI)**	***P*** **value**	**OR (95% CI)**	***P*** **value**
ADHD	1.30 (1.13–1.53)	< 0.001	1.32 (1.13–1.54)	0.001
**Borderline symptoms**				
Inattention	1.48 (1.23–1.77)	< 0.001	1.53 (1.27–1.83)	< 0.001
Hyperactivity/impulsivity	1.31 (1.10–1.56)	0.002	1.31 (1.09–1.55)	0.003
Opposition/defiance	1.51 (1.35–1.67)	< 0.001	1.46 (1.31–1.62)	< 0.001

PCOS, polycystic ovary syndrome; ADHD, attention-deficit/hyperactivity disorder; OR, odds ratio; CI, confidence interval.

* A child was defined as having borderline symptoms on the subset if he/she had at least mild symptoms of inattention (subset score ≥ 13), hyperactivity/impulsivity (subset score ≥ 13), and opposition/defiance (subset score ≥ 8). ADHD was defined if the inattention score ≥ 13 and/or hyperactivity/impulsivity score ≥ 13.

¶ Adjusted for maternal age at childbirth, maternal education, marital status, household income, passive smoking during pregnancy, pre-pregnant body mass index; paternal age at childbirth and paternal education; child sex and child age at ADHD assessment.

### Sex difference in the relationship between maternal PCOS and offspring ADHD at age 3–6 years

Sex-stratified analyses showed a higher odds ratio for ADHD symptoms in boys. Maternal PCOS exposure was associated with increased odds of 1.38 (95% CI: 1.14–1.66), 1.73 (95% CI: 1.38–2.15), 1.35 (95% CI: 1.08–1.66), and 1.55 (95% CI: 1.34–1.78) for ADHD, inattention, hyperactivity/impulsivity, and opposition/defiance symptoms, respectively, in boys after controlling for confounders. Nevertheless, the odds ratio for the girls was attenuated. Although maternal PCOS exposure was still associated with greater odds for opposition/defiance symptoms (1.35, 95% CI: 1.15–1.58), the odds for ADHD (1.23, 95% CI: 0.94–1.57), inattention symptoms (1.21, 95% CI: 0.85–1.66), and hyperactive symptoms (124, 95% CI: 0.90–1.66) became insignificant (Table 3). However, the differences between girls and boys regarding the association between maternal PCOS and offspring ADHD did not reach statistical significance (all *P*-values for interaction >0.05). Similarly, PCOS exposure was associated with higher SNAP scores with a stronger association in boys (adjusted model β = 2.32, 95% CI: 1.65–2.99) than in girls (adjusted model β = 1.45, 95% CI: 0.79–2.12, *P*-value for interaction = 0.063) (Supplementary Table S1).

**Table 3:** Odds of maternal PCOS for ADHD and borderline symptoms in offspring aged 3–6 years stratified by sex^*^.

	**Boys**				**Girls**				***P*** **for interaction**	
	**Crude model**		**Adjusted model** ^**¶**^		**Crude model**		**Adjusted model** ¶		**Crude model**	**Adjusted model^¶^**
	**OR (95% CI)**	***P* value**	**Adjusted model**	***P* value**	**OR (95% CI)**	***P* value**	**OR (95% CI)**	***P* value**		
ADHD	1.38 (1.14–1.65)	0.001	1.38 (1.14–1.66)	0.001	1.22 (0.93–1.56)	0.133	1.23 (0.94–1.57)	0.122	0.450	0.391
**Borderline symptoms**										
Inattention	1.67 (1.33–2.07)	< 0.001	1.73 (1.38–2.15)	< 0.001	1.17 (0.83–1.61)	0.347	1.21 (0.85–1.66)	0.269	0.081	0.066
Hyperactivity/impulsivity	1.36 (1.09–1.67)	0.005	1.35 (1.08–1.66)	0.007	1.24 (0.90–1.66)	0.169	1.24 (0.90–1.66)	0.174	0.643	0.574
Opposition/defiance	1.59 (1.38–1.83)	< 0.001	1.55 (1.34–1.78)	< 0.001	1.41 (1.20–1.65)	< 0.001	1.35 (1.15–1.58)	< 0.001	0.266	0.228

PCOS, polycystic ovary syndrome; ADHD, attention-deficit/hyperactivity disorder; OR, odds ratio; CI, confidence interval.

* A child was defined as having borderline symptoms in the subset if he/she had at least mild symptoms of inattention (subset score ≥ 13), hyperactivity/impulsivity (subset score ≥ 13), and opposition/defiance (subset score ≥ 8). ADHD was defined as an inattention score of ≥13 and/or a hyperactivity/impulsivity score of ≥ 13.

¶ Adjusted for maternal age at childbirth, maternal education, marital status, household income, passive smoking during pregnancy, pre-pregnant body mass index; paternal age at childbirth and paternal education; and child age at ADHD assessment.

### The association of treatment for PCOS with offspring ADHD at age 3–6 years

To determine the impact of PCOS treatment on ADHD risk in children, we performed further analysis by dividing PCOS mothers into treated and untreated groups. As shown in Table 4, our results revealed consistently higher odds of developing ADHD symptoms in children born to untreated PCOS mothers, although the confidence levels generally overlapped between treated and untreated PCOS cases (Table 4). Similar findings were achieved when a SNAP score was used for ADHD severity measurement (Supplementary Table S2).

**Table 4:** Odds of untreated and treated maternal PCOS for ADHD and borderline symptoms in offspring aged 3–6 years^*^.

	**Crude model**		**Adjusted model** ^**¶**^	
	**OR (95% CI)**	***P* value**	**OR (95% CI)**	***P* value**
**ADHD**				
Non-POCS	1.0 (reference)		1.0 (reference)	
Untreated PCOS	1.41(1.08, 1.81)	0.010	1.41 (1.08–1.83)	0.010
Treated PCOS	1.27 (1.06, 1.52)	0.010	1.28 (1.06, 1.54)	0.008
**Inattention**				
Non-POCS	1.0 (reference)		1.0 (reference)	
Untreated PCOS	1.41 (1.12–1.76)	0.002	1.67 (1.20, 2.25)	0.001
Treated PCOS	1.41 (1.12–1.76)	0.002	1.47 (1.17, 1.83)	0.001
**Hyperactivity/impulsivity**				
Non-POCS	1.0 (reference)		1.0 (reference)	
Untreated PCOS	1.52 (1.11–2.01)	0.005	1.51 (1.11, 2.01)	0.006
Treated PCOS	1.22 (0.98–1.51)	0.065	1.22 (0.98, 1.50)	0.074
**Opposition/defiance**				
Non-POCS	1.0 (reference)		1.0 (reference)	
Untreated PCOS	1.71 (1.42–2.05)	< 0.001	1.65 (1.37, 1.98)	< 0.001
Treated PCOS	1.42 (1.25–1.61)	< 0.001	1.38 (1.21, 1.56)	< 0.001

PCOS, polycystic ovary syndrome; ADHD, attention-deficit/hyperactivity disorder; OR, odds ratio; CI, confidence interval.

* A child was defined as having borderline symptoms if he/she had at least mild symptoms of inattention (subset score ≥ 13), hyperactivity/impulsivity (subset score ≥ 13), or opposition/defiance (subset score ≥ 8).

¶ Adjusted for maternal age at childbirth, maternal education, marital status, household income, passive smoking during pregnancy, pre-pregnant body mass index; paternal age at childbirth and paternal education; child sex and child age at ADHD assessment.

## Discussion

This population-based study investigated the association between maternal PCOS and ADHD symptoms in Chinese preschoolers. We confirmed that maternal PCOS was independently associated with ADHD symptoms in offspring aged 3–6 years. Moreover, male offspring tended to be more susceptible to maternal PCOS exposure than female offspring.

Significant associations between maternal PCOS and ADHD symptoms in offspring have been reported (10–16). For instance, three nationwide registry-based cohort studies with thousands of children in Sweden and Finland reported 42–46% greater odds of being diagnosed with ADHD in offspring with maternal PCOS (10, 13, 14). Similarly, a community-based birth cohort study involving 1915 mother-child pairs in upstate New York also reported a similar risk estimate (relative risk = 1.34, 95% CI: 0.86–2.09). However, statistical significance was not achieved, probably due to the small sample size (11). Our study further extends the knowledge of previous studies by demonstrating the intergenerational impact of maternal PCOS on offspring ADHD in Chinese individuals. However, the mechanism underlying this association remains unclear. The sex steroid testosterone may be a crucial link between PCOS and offspring ADHD (29, 30). PCOS is characterized by hyperandrogenism. High levels of prenatal androgen exposure may affect the development of brain regions involved in neurodevelopmental disorders (9). Moreover, women with PCOS have an elevated prevalence of obesity and pregnancy complications, such as gestational diabetes, than women without PCOS (31). These pathophysiological conditions may alter the intrauterine environment and negatively affect the growth of the fetal brain, contributing to the development of ADHD (32). Our study confirmed the association between maternal PCOS and offspring ADHD and reinforced the importance of neurodevelopment screening in children born to mothers with PCOS.

Although boys are two-three times more likely to have ADHD, it remains unclear whether boys are more vulnerable to maternal PCOS exposure than girls. Two Swedish registry-based studies reported a stronger association between maternal PCOS exposure and ADHD in female offspring than in male offspring (10, 13), whereas the Finnish population study reported a similar risk estimate for boys and girls (14). In contrast, the Odense child cohort study involving 1776 mothers reported a positive association (adjusted OR = 2.20, 95% CI: 1.20–4.02) between maternal PCOS and ADHD in male offspring but not in female offspring (adjusted OR = 0.43, 95% CI: 0.13–1.42) (15). Our findings are consistent with those from the Odense cohort study, showing a tendency for a stronger association between maternal PCOS and offspring ADHD in boys than in girls. The underlying causes of the inconsistent tendencies between our study and the studies mentioned above remain uncertain. This might be due to the assessment of ADHD manifestations in the Swedish and Finnish studies (10, 13, 14), in contrast to ADHD symptoms in the Odense child cohort study and the present study. Therefore, further studies are warranted to elucidate the different mechanisms related to the development of ADHD in boys and girls born to mothers with PCOS.

The effect of treatment on the association between maternal PCOS and offspring ADHD remains unknown. The Upstate New York study involving 1915 mother-child pairs reported a lower risk estimate for ADHD in treated PCOS than in untreated PCOS cases; however, the association between PCOS and ADHD was not significant, probably due to the small sample size (11). Likewise, pre-pregnant treatment of PCOS also demonstrated a tendency of reduced risk of developing ADHD in offspring than untreated PCOS, although the confidence levels were overlapping. We hypothesized that this is because PCOS treatment reduces androgen levels and improves insulin resistance in mothers. Such benefits may continue into pregnancy, reducing circulating androgens and inflammatory cytokines, which influence fetal brain development (33–35). However, since we did not have detailed information on the medications used for PCOS treatment, we could not investigate these associations further. Considering the potential effect of PCOS on neurodevelopment in offspring, further studies are necessary to determine which medicines can benefit the offspring.

The major strengths of this study are its population-based design and large sample size. However, there are still some limitations that should be considered when interpreting these findings. First, this study was retrospective and relied on self-reported information; therefore, the results should be treated with caution. Second, the lack of parental history of psychiatric disorders data prevented us from investigating its confounding effects, which might bias our findings (6). Third, since 20% of the parents did not respond to the survey, response bias might exist. However, as a population-based study targeting more than 80,000 preschoolers, an 80% response rate should be excellent enough to conduct the study. Fourth, we did not control for active smoking and alcohol use during pregnancy in the regression model, although smoking and alcohol during pregnancy were uncommon. Fifth, although we tried to differentiate between treated and untreated PCOS cases, we did not collect information on the therapy methods, preventing us from further investigating the association between PCOS treatment and offspring ADHD. Lastly, we did not collect information on the phenotypes of PCOS, which can be divided into A-D (four phenotypes) according to the clinical manifestation. Therefore, we could not conclude the relationship between the specific phenotype and the ADHD symptoms in offspring.

In conclusion, maternal PCOS is associated with a higher risk of ADHD in offspring, with boys being more susceptible to the exposure than girls. Further studies are needed to confirm our findings, and early neurodevelopmental screening may be needed in children born to mothers with PCOS.

## Data availability statement

The raw data supporting the conclusions of this article will be made available by the authors, without undue reservation.

## Ethics statement

The studies involving human participants were reviewed and approved by the Ethics Committee of Shenzhen Longhua Maternity and Child Healthcare Hospital. Written informed consent to participate in this study was provided by the participants' legal guardian/next of kin.

## Author contributions

YuZ and DL researched data, wrote, and edited the manuscript. VG, SQ, JZ, and YaZ contributed to the data collection and review of the manuscript. YW and WC contributed to the discussion, reviewed, and edited the manuscript. BW and WY conceptualized the study, contributed to the discussion, and reviewed and finalized the manuscript. All authors have read and approved the final manuscript.
