# Supplementary material for: Association between maternal polycystic ovary syndrome and attention-deficit/hyperactivity disorder in offspring aged 3–6 years: A Chinese population-based study
Source: Front Public Health. 2023 Jan 9;10:1032315. doi: 10.3389/fpubh.2022.1032315 (PMC9868860; doi:10.3389/fpubh.2022.1032315)
Supplement: Supplementary file 1 [file Table_1.DOCX]

**Table S1. Associations of maternal PCOS with SNAP-IV total score in offspring aged 3-6 years.**

|  | Total | |  | Boys | |  | Girls | | P for interaction |
| --- | --- | --- | --- | --- | --- | --- | --- | --- | --- |
|  | β (95%CI) | P value |  | β (95%CI) | P value |  | β (95%CI) | P value |  |
| Crude model | 1.94 (1.46-2.42) | <0.001 |  | 2.34(1.66-3.01) | <0.001 |  | 1.48 (0.81-2.16) | <0.001 | 0.080 |
| Adjusted model¶ | 1.91 (1.44-2.39) | <0.001 |  | 2.32(1.65-2.99) | <0.001 |  | 1.45 (0.79-2.12) | <0.001 | 0.063 |

PCOS, polycystic ovary syndrome; CI, confidence interval.

**¶**Adjusted for maternal age at childbirth, maternal education, marital status, household income, passive smoking during pregnancy, pre-pregnant body mass index; paternal age at childbirth and paternal education; child sex (in the total samples) and child age at ADHD assessment.

**Table S2. Associations of treated and untreated maternal PCOS with SNAP-IV total score in offspring aged 3-6 years.**

|  | Crude model | |  | Adjusted model¶ | |
| --- | --- | --- | --- | --- | --- |
|  | β (95%CI) | P value |  | β (95%CI) | P value |
| Non-PCOS | 1.0 |  |  | 1.0 |  |
| Untreated PCOS | 1.93 (1.07-2.78) | <0.001 |  | 1.89 (1.05-2.73) | <0.001 |
| Treated PCOS | 1.94 (1.36-2.52) | <0.001 |  | 1.92 (1.36-2.49) | <0.001 |

PCOS, polycystic ovary syndrome; CI, confidence interval.

**¶**Adjusted for maternal age at childbirth, maternal education, marital status, household income, passive smoking during pregnancy, pre-pregnant body mass index; paternal age at childbirth and paternal education; child sex and child age at ADHD assessment.
